# Efficacy of Potassium Iodide and Glutathione for Correlation of Dentin Discoloration Caused by Silver Diamine Fluoride

**DOI:** 10.7759/cureus.68498

**Published:** 2024-09-03

**Authors:** Mahsa Samani, Hamid Majzoub, Faramarz Zakavi, Ayyub Mojaddami

**Affiliations:** 1 Department of Restorative Dentistry, Faculty of Dentistry, Ahvaz Jundishapur University of Medical Sciences, Ahvaz, IRN; 2 Department of Medical Chemistry, Faculty of Pharmacy, Ahvaz Jundishapur University of Medical Sciences, Ahvaz, IRN; 3 Toxicology Research Center, Medical Basic Sciences Research Institute, Ahvaz Jundishapur University of Medical Sciences, Ahvaz, IRN

**Keywords:** dentin, discoloration, glutathione, potassium iodide, silver diamine fluoride

## Abstract

Objective: This study aimed to investigate and contrast the degree of dentin discoloration resulting from the application of silver diamine fluoride (SDF) alone, SDF in combination with potassium iodide (KI), and SDF in combination with glutathione. The aim was to assess the effectiveness of these combinations in reducing the aesthetic issues associated with SDF treatment in minimally invasive dentistry and preventive procedures.

Methodology: We conducted this in-vitro study on 136 permanent molar teeth. The teeth were randomly assigned to two main groups of total-etch and self-etch and four subgroups of control, SDF, SDF plus KI, and SDF with glutathione. The teeth underwent colorimetry before and 1 week after applying materials to compare the color change. We mounted the teeth in acrylic resin 1 mm below their cementoenamel junction. A low-speed diamond saw and 600-grit silicon carbide abrasive paper eliminated the occlusal enamel for 10 seconds under water coolant, exposing the dentin. The teeth were then immersed in a demineralizing solution (pH of 4.4, 50 mmol acetate, 2.2 mmol potassium dihydrogen phosphate, and 2.2 mmol calcium chloride) for 7 days at 37°C to induce dentin demineralization and simulate demineralized dentin artificially. The teeth were then gently polished with 600-grit silicon carbide abrasive paper to create a standard smear layer and eliminate the demineralized dentin layer.

Results: 136 teeth were evaluated in the total-etch (n = 68) and self-etch (n = 68) groups. Glutathione and KI significantly decreased the discoloration caused by SDF (P<0.05). No significant difference was noted in color change between SDF/glutathione and SDF plus KI groups (P > 0.05). In self-etch groups, glutathione yielded an ∆E=4.12, while KI yielded an ∆E=4.44, with no significant difference.

Conclusion: The application of glutathione and KI can significantly decrease dentin discoloration caused by SDF.

## Introduction

Dental caries, commonly called dental decay, is a complicated oral disease that arises from an imbalance between the tooth’s demineralization and remineralization processes [1]. Margolis and Moreno underlined tooth plaque’s role in the development of caries. When the researchers exposed the tooth to fermentable carbohydrates, they observed a dramatic rise in demineralization and a rapid reduction in the amount of calcium phosphate in the plaque. The primary explanation for this occurrence was the production of lactic acid and the reduction of plaque fluid, which led to caries development [2]. Industrialized and developing nations frequently see dental caries, a common chronic condition in children [3]. Untreated dental caries affect children in developing countries because of their low socioeconomic status, limited access to basic oral hygiene, high dental costs, and lack of resources [4].

The traditional method for managing dental caries entails removing the demineralized dentinal structure and replacing it with a substance compatible with the body. Treating deep dentinal caries can be challenging due to the need for expensive equipment, advanced dental expertise, and patient cooperation. Managing dental caries in young children poses a challenge for clinicians due to their limited ability to handle the situation [5]. Exploring various strategies to alter biofilm effectively is crucial for enhancing oral health. Improving the process of tooth structure remineralization can help reduce dental caries. This leads to a fundamental alteration in managing and preventing dental caries. Dentists need more widely performed dental procedures that are affordable, safe, effective, and flexible enough to treat high-risk patients in various clinical settings [6].

Silver diamine fluoride (SDF) effectively halted the development of tooth decay in primary and early permanent teeth in children, as well as stopping the progression of root decay in older patients. It was first approved for therapeutic use in Japan during the 1960s. From the 1960s until the 1990s, countries including Argentina, Australia, Brazil, and China employed SDF as a preventive treatment to stop the progression of dental caries. Moreover, practitioners have employed SDF to manage dentinal hypersensitivity. However, the Food and Drug Administration (FDA) authorized the first SDF agent in commerce in August 2014 [1]. Zhao et al. state that SDF is a transparent solution that kills bacteria and enhances the restoration of demineralized tooth structures [7].

Nevertheless, the primary drawback of SDF is the staining of tooth enamel and dentin, which consequently limits its practical application due to concerns about its appearance. Knight et al. have recommended using KI to decrease discoloration [8]. The potassium iodide (KI) crystals undergo a reaction with the SDF’s free silver ions, resulting in the formation of a creamy white precipitate. This precipitate aids in lessening the discoloration that SDF causes. Glutathione (GSH) has recently emerged as a substitute for KI. When combined with SDF, GSH effectively maintains the presence of silver ions both within SDF and on the tooth substrate. In the past, GSH has facilitated interactions among intricate biosystems [8]. Therefore, this study aimed to assess and compare the impact of KI and GSH on tooth discoloration following the use of 30% SDF in permanent molars. The primary goals were to assess the impact of KI and GSH separately and then compare their effects on tooth discoloration following the application of 30% SDF to permanent molars.

## Materials and methods

This in-vitro experimental study was conducted on extracted sound molar teeth with no restorations or caries collected from different dental clinics and offices in Ahvaz City. The teeth were stored in 0.1% thymol (Sigma Aldrich) immediately after extraction. All teeth evaluated in this study had been extracted within the past 6 months. The Ahvaz Jundishapur University of Medical Sciences ethics committee approved the study. The teeth were mounted in acrylic resin to 1 mm below their cementoenamel junction. The occlusal enamel was eliminated by a low-speed diamond saw and 600-grit silicon carbide abrasive paper for 10 seconds under water coolant to expose dentin. The teeth were then immersed in a demineralizing solution (pH of 4.4, 50 mmol acetate, 2.2 mmol potassium dihydrogen phosphate, and 2.2 mmol calcium chloride) for 7 days at 37°C to induce dentin demineralization and simulate demineralized dentin artificially. The teeth were then gently polished with 600-grit silicon carbide abrasive paper to create a standard smear layer and eliminate the demineralized dentin layer. Each tooth was only used for one test. The teeth were then randomly divided into eight groups (n = 17) (Table 1).

**Table 1:** Group properties SDF: silver diamine fluoride; SDF + KI: silver diamine fluoride plus potassium iodide; SDF + glutathione: silver diamine fluoride plus glutathione

Teeth Group	Group Properties
Group 1	Total-etch control group
Group 2	SDF with total-etch bonding agent
Group 3	SDF + KI with total-etch bonding agent
Group 4	SDF + glutathione with total-etch bonding agent
Group 5	Self-etch control group
Group 6	SDF with self-etch bonding agent
Group 7	SDF + KI with self-etch bonding agent
Group 8	SDF + glutathione with self-etch bonding agent

The specimens underwent colorimetry before applying materials to assess their baseline color.

Bonding agents, including Adper Single Bond 2 (3M) total-etch adhesive and Clearfil SE Bond 2 self-etch adhesive (Kuraray Noritake Dental Inc.) and 30% SDF (Cariestop Biodinamica) were used according to the manufacturer’s instructions. In groups 1 and 5, dentin was rinsed with saline. In groups 2 and 6, one drop of SDF was applied by a micro brush to dentin surfaces and rinsed after 90 seconds. In groups 3 and 7, after the application of SDF, KI (Merck, Germany) was applied by a micro brush until a white deposit formed and was then rinsed for 90 seconds. In groups 4 and 8, similar to groups 3 and 7, glutathione (Merck, Germany) was used instead of KI. The color of the dentin surface was assessed again by a spectrophotometer. In groups 1 to 4, after etching with 37% phosphoric acid, a single-bond total-etch bonding agent was applied by a microbrush. In groups 5 to 8, as instructed by the manufacturer, SE Bond primer was first applied, and then adhesive was used.

The color of dentin specimens was measured after immersion in artificial saliva for one day. The color of the dentin surface was measured by a spectrophotometer (JP7200F; Juki, Tokyo, Japan). The CIE L*a*b* color system was used for the 3D assessment of color by measuring the L*, a*, and b* color coordinates. The color change (∆E) was calculated using the following formula:

∆E = [(∆L)2 + (∆a)2 + (∆b)2]1/2

The threshold of perception of color change for the naked eye was considered ∆E > 3.7 units [9].

The Kolmogorov-Smirnov test was used to assess the normality of the data distribution. For the normally distributed data, a one-way ANOVA was used to compare quantitative variables, while the Chi-square test was used to compare qualitative variables. Non-parametric tests were used for non-normally distributed data. All statistical analyses were performed by SPSS version 24 at the 0.05 significance level.

## Results

**Figure 1:**
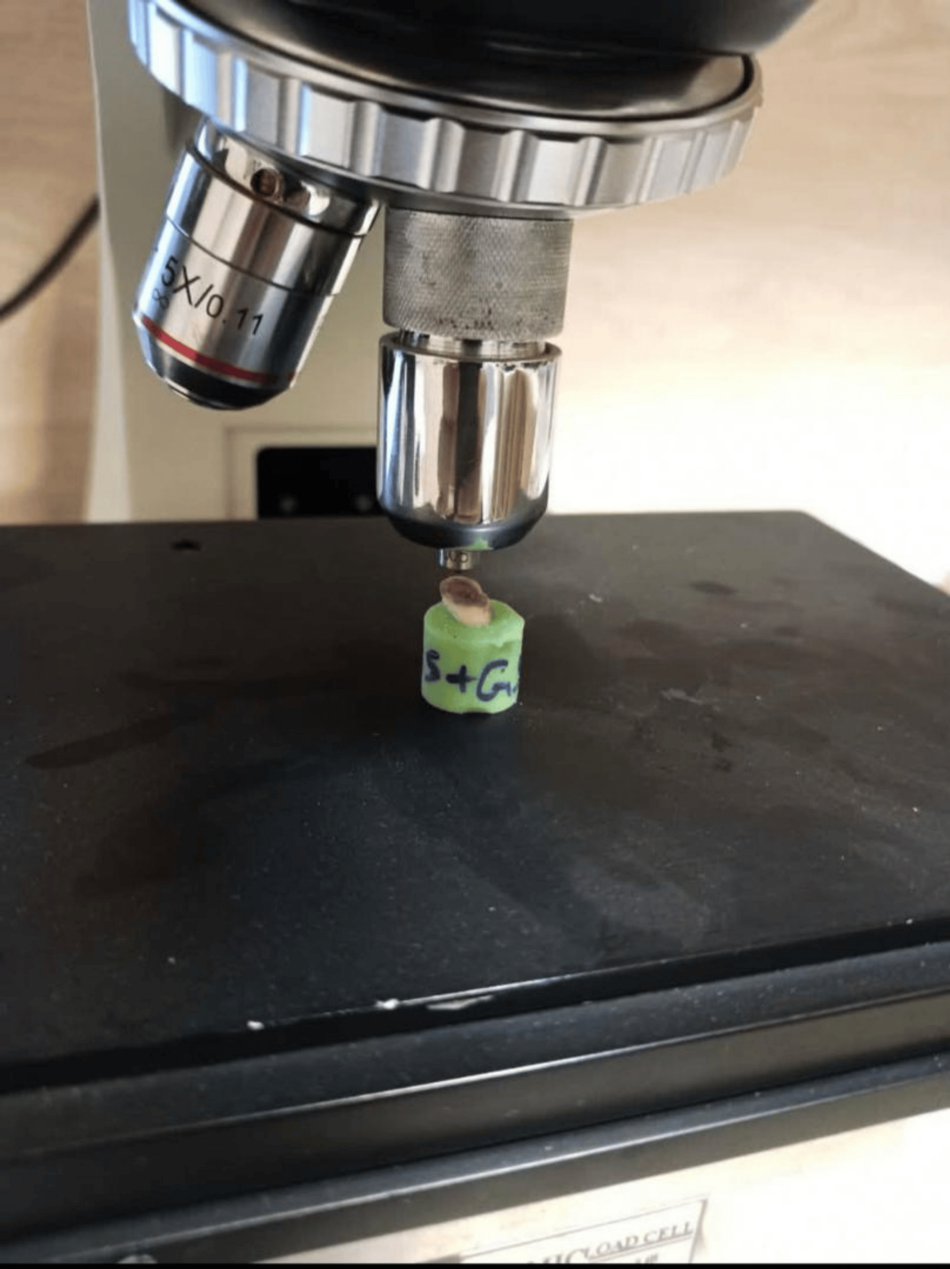
Assessment of color change

**Figure 2:**
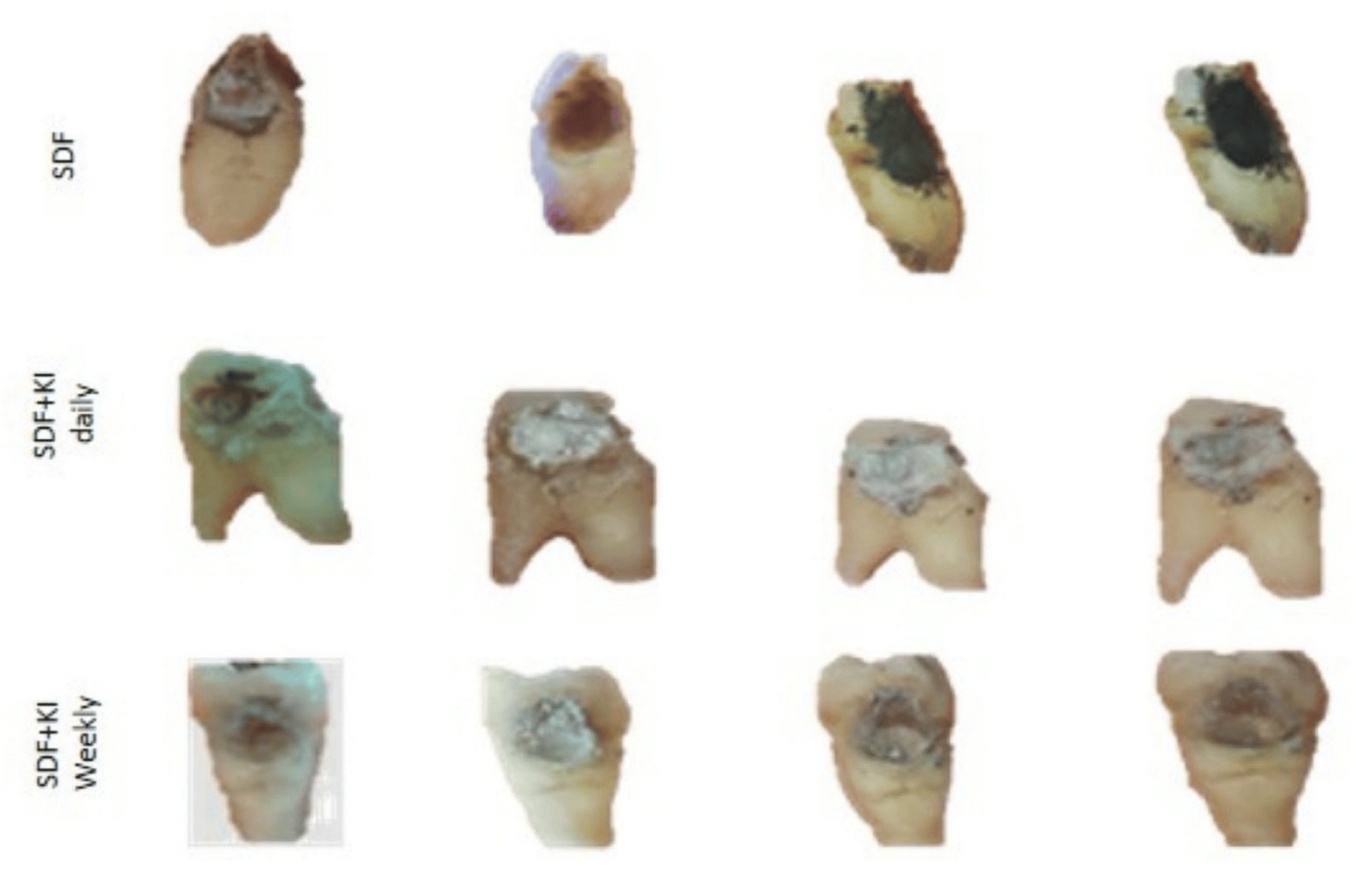
Images at different follow-up intervals

Considering the darkening of color after the application of SDF, the selected color spectrum for the comparison ranged from white to black (white: 255, black: 0). The gray value index was used as the average gray color in this spectrum. The higher the gray value, the whiter the color, and vice versa. The ∆E of the mean gray value indicates the mean difference of the gray value before and after using KI and glutathione (Figures 1-2).

As shown in Table 2, the gray value after using KI or glutathione approximated the color in the control group. It means that applying KI and glutathione decreased the discoloration caused by SDF. 

**Table 2:** ∆E comparison in Total-etch and Self-etch groups and their subgroups

Groups		∆E Mean ± SD	Minimum ∆E	Maximum ∆E	Total (N)
Total-etch	Control	1.3 ± 0.36	5.1	9	10
	Silver diamine fluoride	11.87 ± 2.32	4	15	15
	Silver diamine fluoride plus potassium iodide	5.86 ± 1.53	0.98	9	9
	Silver diamine fluoride plus Glutathione	4.32 ± 2.4	1.32	8	17
Self-etch	Control	1.1 ± 0.89	4.3	8.6	13
	Silver diamine fluoride	12.3 ± 3.56	9	16.53	15
	Silver diamine fluoride plus potassium iodide	4.44 ± 2.01	3	5.3	12
	Silver diamine fluoride plus glutathione	4.12 ± 1.76	1.38	9.71	17

Considering the color change after the application of SDF, the maximum ∆E was recorded in the SDF group 7 days after restoration. In total-etch groups, the performance of GSH was better than that of KI, but not significantly. In self-etch groups, glutathione yielded an ∆E=4.12, while KI yielded an ∆E=4.44, with no significant difference (Table 3). 

**Table 3:** ∆E mean and std. deviation (maximum-minimum) in total-etch and self-etch groups and their subgroups

Groups	Control	Silver diamine fluoride	Silver diamine fluoride plus potassium iodide	Silver diamine fluoride plus glutathione	p-value
Total-etch	1.3 ± 0.36	11.87 ± 2.32	5.86 ± 1.53	4.32 ± 2.4	<0.001
Self-etch	1.1 ± 0.89	12.3 ± 3.56	4.44 ± 2	4.12 ± 1.76	<0.001

## Discussion

The application of SDF for treating dental caries has long been of interest due to its optimal strengthening effect and antimicrobial activity, leading to its popularity. The presence of high concentrations of fluoride (44,880 ppm) and silver (25.5%) in the composition of SDF enables its reaction with dental structures and the subsequent release of considerable amounts of silver ions (Ag+). The application of SDF is a non-invasive, affordable, and simple technique for managing caries [9]. Following the application of SDF to the tooth structure, it reacts with the tooth and releases high amounts of silver ions. With a reduction in the release of silver ions over time, they accumulate deposits and form dark spots, which are mainly due to the formation of silver phosphate.

Silver phosphate crystals are yellow at first but become dark following exposure to light and other chemical agents [2]. The antibacterial activity of SDF is based on its action method against cariogenic bacteria. Additionally, SDF promotes the remineralization of carious dentin, inhibits the demineralization of enamel and dentin, and protects collagen from degradation in demineralized dentin [10,11]. Dark discoloration of areas restored with SDF forced the researchers to find some materials or solutions as an additive to prevent discoloration while preserving the consistency and antibacterial activity of SDF. KI and glutathione are bioactive agents with increasing popularity for use in combination with SDF. KI reacts with free silver ions and forms silver iodide, which creates a creamy white deposit [12]. It decreases the availability of silver ions and improves the color as such [13, 14]. Glutathione was used on silver particles as a biomimetic coating to reinforce reactions with complex biosystems and increase their solubility in water [15]. It is a tri-peptide biomolecule that has a high affinity for metal ions due to having thiol (SH-) groups. Glutathione covers the silver particles and prevents discoloration by decreasing their accumulation. This study aimed to assess the efficacy of KI and glutathione for correcting dentin discoloration caused by SDF. It appears that KI binds to the free silver ions in SDF and prevents the formation of silver phosphate, which is responsible for the discoloration caused by SDF and creates a yellow deposit of silver iodide.

In assessing color change after the application of SDF, glutathione showed slightly superior results than KI in the present study, although this difference was not significant (4.12 versus 4.44). Sayed et al. [13] observed changes in dark and light conditions. Because color change due to silver phosphate deposition occurs gradually in the tooth structure, the color change was evaluated after 3, 6, 24, 48, and 72 hours, and 7, 10, and 14 days after its application in their study. Their results showed that KI and glutathione significantly decreased discoloration. The difference between KI and glutathione was insignificant at 2 and 6 hours in their research, which was in line with the present results. However, at 10 and 14 days, KI had superior performance compared with glutathione, but not significantly.

In contrast, glutathione performed better than KI in the present study. This difference arises because we compared both groups once after the intervention in the present study, and the difference between KI and glutathione in color correction was not significant, which was in line with the results of Sayed et al. [13]. Gupte et al. [16], in their study conducted in 2021 in the United States, compared the efficacy of glutathione and KI after applying SDF. Unlike the present study, they did not have a control group. They compared three groups (n = 15) of SDF, SDF plus KI, and SDF plus glutathione. They measured the lightness and two chromaticity parameters before and after the procedure. Assessment of color change at different time points was the main strength of their study since they evaluated the color of specimens after 5 minutes, 24 hours, and 1, 2, and 4 weeks following the application of SDF. The groups that used SDF plus KI and SDF plus glutathione did better than the SDF group after 24 hours, which was in line with what we found here.

In recent years, researchers have explored several methods for the color assessment of specimens, such as shade guides, calorimeters, and digital analysis of images by software.

Detsomboonrat et al. [17] (2022) tried to find the most appropriate concentration of KI to reduce dark spots caused by SDF on extracted carious teeth. They found that applying KI significantly decreased the dark areas in a dose-dependent manner. However, during 14 days, the difference in reduction of spots by different doses of KI was minimal. Sorkhdini et al. [18] (2020) indicated the significant efficacy of KI in combination with SDF for removing primary enamel discolorations. The discoloration caused by SDF in the tooth structure does not decrease with pH cycling, indicating the durability of the dark spots against repeated acid attacks induced by pH cycling. Their results agreed with the present findings, although we used dentin specimens and did not perform pH cycling. Vennela et al. [19] evaluated the primary carious teeth and concluded that applying KI before tooth-colored restorations minimized the discoloration caused by SDF under such repairs. Hamdy et al. [20] conducted a study to assess the ability of different materials to mask the discoloration caused by SDF in primary teeth. They found that immediately after applying SDF, all tested materials, including KI, universal composite, and glass ionomer, effectively masked the discoloration caused by SDF. However, the composite resin was the only material whose masking effect was unaffected by the aging process. KI also showed optimal masking ability. Thus, further studies are required to assess the masking ability of KI in combination with composite resin.

Patel et al. [21] evaluated primary molar teeth and reported results in conformity with the present findings, indicating the optimal efficacy of SDF and KI in reducing discolorations compared with SDF alone. However, they used digital image analysis software in their study. Although the digital technique appears more accurate, it is not routinely used to assess tooth color.

Following irrigation, SDF does not leave any residues on dentin surfaces. However, SDF particles can penetrate dentin due to their small size. Silver and fluoride ions can penetrate 450 µm deep in relatively demineralized dentin. Also, silver ions released from SDF have a high affinity for collagen proteins. Thus, they penetrate deep dentin containing exposed collagen. SDF granules reach a depth of 30 µm into the dentinal tubules, and after SDF is applied, a layer of silver phosphate forms inside the dentin. SDF penetrates dentinal tubules, and forms sound inter-tubular dentin. In the present study, dentin was demineralized, and SDF penetrates deeper into demineralized dentin [18-20].

Limitations of the study

The study used extracted teeth in a controlled laboratory setting, which might not accurately replicate the complex oral environment and physiological processes present in living things. The assessment of color change was carried out only one week after the chemicals were applied. Longer-term observation would provide a more thorough understanding of how long-lasting the color change is and whether it can be undone.

## Conclusions

Ultimately, our research shows that using KI, glutathione, and SDF together significantly reduces dentin discoloration compared to using SDF alone. The results suggest that preventive and minimally invasive dentistry might be able to use these compounds to help with the cosmetic problems that come with SDF therapy. Nevertheless, further clinical investigations are necessary to validate these results and evaluate their practicality in dental settings.
